# instaGraminoid, a Novel Colorimetric Method to Assess Herbicide Resistance, Identifies Patterns of Cross-Resistance in Annual Ryegrass

**DOI:** 10.34133/2019/7937156

**Published:** 2019-04-23

**Authors:** Jefferson F. Paril, Alexandre J. Fournier-Level

**Affiliations:** School of Biosciences, University of Melbourne, Parkville, VIC, Australia

## Abstract

Herbicide resistance in agricultural weeds is a global problem with an increasing understanding that it is caused by multiple genes leading to quantitative resistance. These quantitative patterns of resistance are not easy to decipher with mortality assays alone, and there is a need for straightforward and unbiased protocols to accurately assess quantitative herbicide resistance.* instaGraminoid*—a computer vision and statistical analysis package—was developed as an automated and scalable method for quantifying herbicide resistance. The package was tested in rigid ryegrass (*Lolium rigidum*), the most noxious and highly resistant weed in Australia and the Mediterranean region. This method provides quantitative measures of the degree of chlorosis and necrosis of individual plants which was shown to accurately reflect herbicide resistance. We were able to reliably characterise resistance to four herbicides with different sites of action (glyphosate, sulfometuron, terbuthylazine, and trifluralin) in two* L. rigidum* populations from Southeast Australia. Cross-validation of the method across populations and herbicide treatments showed high repeatability and transferability. Significant positive correlations in resistance of individual plants were observed across herbicides, which suggest either the accumulation of herbicide-specific resistance alleles in single genotypes (multiple stacked resistance) or the presence of general broad-effects resistance alleles (cross-resistance). We used these quantitative estimates of cross-resistance to simulate how resistance development under an herbicide rotation strategy is likely to be higher than expected.

## 1. Introduction

Weeds are a major issue in modern cropping systems with yield losses due to infestation ranging from 7.5% to 10.5% in important crops, i.e., wheat, rice, maize, potato, soybean, and cotton [1, 2]. This typically represents a revenue loss of $708 million per year in Australia alone [3]. Herbicide application is the most effective means to control weeds in broad-acre cropping [4], and the intense selection pressure imposed by herbicides has driven the evolution of resistance in multiple weed species. According to the International Survey of Herbicide Resistant Weeds as of 2018, there are 495 unique reported cases of herbicide resistance spanning 254 species [5]. This trend shows that herbicide application strategies over the past four decades is not sustainable and will eventually result in the complete loss of herbicide efficacy.

Maintaining the efficacy of herbicides requires active management of resistance in weed populations. The emergence of herbicide resistance in the field must be detected early and its development must be monitored. This is particularly critical as early detection of resistance can trigger the deployment of efficient preemptive strategies instead of reactive ones. The most widely adopted resistance assays measure mortality by scoring the number of dead and surviving plants after herbicide application. These methods generate useful and unambiguous binary data characterising levels of resistance; however, mortality assays may not be sensitive enough to reveal early signs of resistance that could be helpful for preemptive strategies. Resistance is increasingly found to be polygenic [6], due to the combination of multiple target-site resistance (TSR) mechanisms (e.g., mutation or overexpression of the target protein) together with non-target-site resistance (NTSR) mechanisms (e.g., detoxification and sequestration mechanisms) [7–10]. Thus, measuring herbicide resistance of individual plants on a quantitative scale can provide the precision needed to finely monitor the development of resistance. This also enables a better capture of the underlying genetic nature of resistance by increasing the statistical power to detect small effects and eventually a better prediction of resistance in field populations.

Quantifying herbicide resistance and overall plant health can be approached in different ways, e.g., visual inspection, visible light imaging, chlorophyll fluorescence imaging, and hyperspectral imaging [11, 12]. Visual inspection is the least costly but can be highly biased, inaccurate, and low-throughput. Image-based approaches are objective and therefore more accurate and potentially high-throughput.

Visual inspection at the population-level described in [9, 13–18] does not generate the level of precision required to monitor individual plant resistance. Furthermore, these require considerably larger areas to replicate whole populations compared with individual plant assays. In addition, using heterogeneous groups of individuals as the experimental unit does not allow for one-to-one genotype comparisons. Individual-level phenotyping through visual inspection [19] can be highly subjective and nontransferable. Assays performed* ex situ*, i.e., in agar [20, 21] and excised leaf assays [22, 23] minimise the confounding effects of environmental factors and reduce the amount of space required but at the expense of accounting for the effects of edaphic factors and whole plant response in the case of excised leaf assays.

Hyperspectral imaging techniques (e.g., normalised difference vegetation index (NDVI) and chlorophyll fluorescence and thermal imaging, as well as near-infrared (NIR) and Raman spectroscopy [11, 13, 14, 24–28]) are capable of highly objective and transferable characterisation of individual plants. Raman spectroscopy has been used to distinguish abiotic stresses in plants [28]. Imaging pulse amplitude modulated (PAM) fluorometry with the purposely built Weed PAM [13, 14, 24, 25] has been used to assess herbicide resistance of weed seedlings in greenhouse conditions [29] and in the field [14]. However, hyperspectral imaging techniques require substantial investment to purchase highly specialised equipment which may not be economical for small laboratories and farmers interested in simple fine-level monitoring of herbicide resistance.

The scale and magnitude of the herbicide resistance crisis motivate the need for accurate and repeatable bioassays with high accuracy and repeatability, yet relying on basic equipment available to the broadest possible user base [30]. Hence, visible light imaging can provide a balance between cost, accuracy, and throughput. The xenobiotic effects of herbicides cause leaf senescence which progresses from chlorosis to necrosis, as well as growth stunting and eventually death of susceptible plants, while resistant plants remain green, growing, and healthy [31, 32]. These effects can be captured simply and objectively using digital imaging in the visible light spectrum. Leaf senescence and overall plant health have been accurately measured and monitored in real-time using coloured images of plant leaves under the visible light spectrum [33, 34]. This implies that it is possible to assess plant health from coloured photographs through colour correction, deblurring for low resolution images [33], followed by image segmentation to isolate the leaves from the background. Morphological characterisation and colour-based metrics can then be calculated using either RGB (red, green, blue) or HSV (hue, saturation, value) colour models [11, 32–34].

Here we develop a novel RGB-based colorimetric method to compute multiple quantitative measures of herbicide resistance for individual plants. Photographs taken at discrete time points and the rates of change over time were used to perform optimal feature selection using* elastic-net *to train a model for predicting individual plant mortality to herbicides. We highlight a potential application of the quantitative resistance estimates by generating predictions about resistance development under four herbicide rotation strategies.

## 2. Materials and Methods

### 2.1. Plant Materials and Herbicide Application

Ryegrass population samples were collected from two cropping areas located 369 km apart in Southeast Australia. The first population was sampled from different field crops (canola, wheat, and barley) on a single farm in Inverleigh, Victoria, while the second population was from a barley field in Urana, New South Wales. Seeds were stored at room temperature for six months to release dormancy and germinated in 0.8% agar at 15°C/25°C night/day temperatures with 12-hour photoperiod and 50% relative humidity. Each seedling was transferred to individual pots with soil media composed of 55% peat moss, 15% perlite, 5% vermiculite, and 25% sand (dry volume). For each population, approximately 500 plants were maintained. Thirty days after transplanting, each plant was cloned by separating four tillers, taking care to include roots or root primordia during transplanting. Fourteen days after cloning through tiller separation, each set of clones was exposed to one of the four herbicides (Table 1). Herbicides were applied at the recommended field rates for a total volume corresponding to 200L*∙*ha^−1^ to simulate field application.

### 2.2. Phenotype Acquisition and Image Analysis

**Figure 1:**
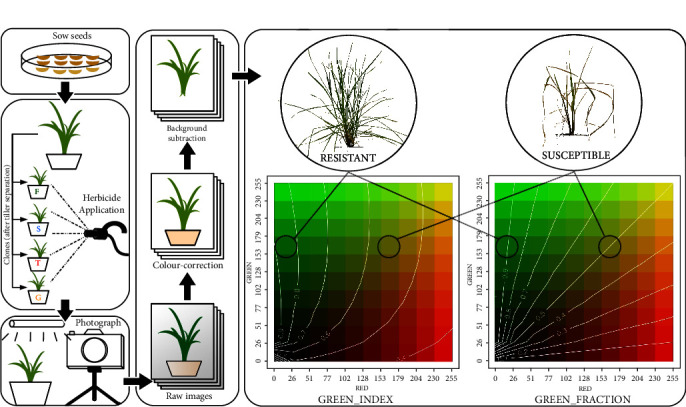
Workflow using instaGraminoid to extract the quantitative herbicide resistance estimates from plant photographs.

For each individual plant, above-ground plant tissues were photographed 0, 7, and 14 days after herbicide application from 4 perpendicular angles (each angle was treated as a technical replicate) under well and uniformly lit condition in a light box (dimensions: 61×61×61 cm; 2 light bands with 30 light emitting diodes each for a total of 13,000 lumens) with a fixed reference white background using a Nikon D7000 camera at 4,928 × 3,264 pixels resolution in “Auto” mode without camera-flash. Survival was scored 21 days after herbicide application. The photographs were colour-corrected by scaling the pixel values in the red, green, and blue channels with the correction factor w_0_/*μ*_i_ where w_0_ is the expected pixel value of the white background and *μ*_i_ is the observed mean pixel value of the white background in the i^th^ colour channel. The colour-corrected images were transformed into grayscale and then into a binary image using the user-defined threshold value (threshold value was set at 75% of w_0_). Morphological transformations with a 5x5 kernel were used to improve the detection of the thin leaves. Object detection using topological structural analysis [35] was used to isolate the above-ground plant tissues from the background. Leaf area, 15 leaf colour-based metrics, and change over pairs of measurements or deltas (Table 2) were calculated from pixel information of the colour-corrected and background-subtracted photographs. This workflow is depicted in Figure 1.

### 2.3. Model Construction and Statistical Analysis

To estimate herbicide resistance quantitatively, a logistic regression model for herbicide mortality was built on the 102 features (51 individual time points plus 51 deltas) using the R/glmnet package. The binary survival data *y*_*i*_ ∈ {0,1} was modelled using a logistic function,

(1) $$\begin{array}{c} p\left(x_{i}\;|\;y_{i}\right)=\frac{1}{1+e^{-\left(\beta_{0}+{x^{\prime }}_{i}\beta \right)}}, \end{array}$$

where **x**_*i*_ are the metrics extracted from the plant photographs and **β** is the regression coefficient associated with each metric. This logistic regression model was fitted to the mortality data using elastic-net penalisation [36],

(2) $$\begin{array}{c} \underset{\beta }{\mathrm{argmin}}-\left[\frac{1}{N}\overset{N}{\underset{i=1}{\sum }}y_{i}\left(\beta_{0}+{x^{\prime }}_{i}\beta \right)-\log\left(1+e^{\left(\beta_{0}+x_{i}^{\prime }\beta \right)}\right)\right]+\lambda \left(\frac{1-\alpha }{2}{\left\|\beta \right\|}_{2}^{2}+\alpha {\left\|\beta \right\|}_{1}\right), \end{array}$$

where the first term is the loss function of the logistic model given the observed mortality data, **y**, while the second term is the elastic-net penalty controlled by *α* and the tuning parameter* λ*. The *α* value was set to 0.5. The *λ* value was selected from a range of 100 values with* λ*_*min*_ = 0.0001 *λ*_max_ at all coefficients equal to zero, by minimising the residual variance in the training set. The quantitative resistance ${\hat{y}}_{i}$ was computed as the standardised negative exponent of* e*, *β*_*0*_* + ***β**′**x**_*i*_ from the logistic equation. Metrics that were given nonzero **β** coefficients are hereafter termed features, consistent with the machine learning literature.

The performance of the* instaGraminoid* method in assessing plant resistance was measured as the proportion of individuals correctly classified as resistant if $1/\left(1+e^{-\hat{y_{i}}}\right)\geq 0.5$ or as susceptible if $1/\left(1+e^{-\hat{y_{i}}}\right)<0.5$. Transferability across populations and herbicide was assessed through 10-fold cross-validation by training the logistic model on a subset of the data (i.e., one population across herbicides, one herbicide across populations, and one population for one herbicide) and testing the predictive ability of the model on a different subset. Each validation set was randomly divided into 10 subsets, and the predictive ability of the trained model was estimated on each of these subsets. This was repeated for 10 iterations to get 100 performance estimations for each training-validation set pair. Correlations between resistance levels to herbicide treatments using Pearson's correlation coefficient (*ρ*) were calculated for each pair of herbicides. The receiver operating curve (ROC) plot was used to visualise the true positive discovery rate as the false positive rate increases.

**Figure 2:**
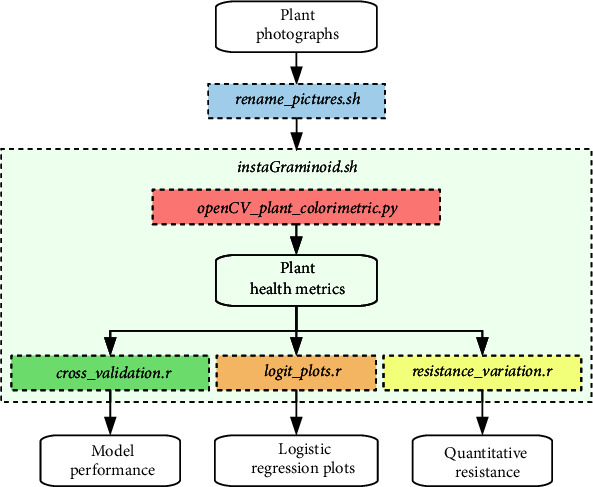
Architecture of the instaGraminoid package.

The resistance characterisation workflow (Figure 1), including the software (Figure 2) designed to extract the quantitative herbicide resistance trait, is packaged into an open source project called* instaGraminoid* (https://gitlab.com/jeffersonfparil/instaGraminoid.git. Standalone executables for Mac OS (tested in OS X High Sierra), Microsoft Windows (tested in Windows 10), and Linux (tested in Ubuntu 18.04) can be found in https://gitlab.com/jeffersonfparil/instaGraminoid_executables.git). These programs were written in Bash 4.3 [37] using GNU-parallel version 2016 [38], Python 2.7 [39], using the Open Source Computer Vision (OpenCV 3.4) [40] and Scientific Python libraries (numpy 1.14 and scipy 1.0) [41], R 3.4 [42], using the glmnet 2.0 [36] and ROCR 1.0 [43] packages.

### 2.4. Herbicide Resistance Development Simulation Based on Resistance Correlation

The development of herbicide resistance under different scenarios was simulated based on the observed resistance levels and the correlations in resistance between the different herbicides using a quantitative genetics framework. The quantitative resistance estimates determined experimentally were scaled to range from 0 for maximum susceptibility to 1 for maximum resistance. Additive genetic variance (*σ*^2^_a_) was modelled as a parabolic function of resistance level, with maximum variance at the intermediate resistance level of 0.5 and minimum at the extreme resistance levels of 0 and 1. The evolution of resistance was simulated based on phenotypic variance (*σ*^2^_p_) and additive genetic variance with the resulting heritability (*σ*^2^_a_/*σ*^2^_p_) set to a maximum of 80% and assuming an infinite population size. Selection for herbicide resistance was applied every generation for 12 generations using the multivariate breeder's equation [44]:

(3) $$\begin{array}{c} \Delta z=G\beta , \end{array}$$

where Δ**z** is the vector of response to selection;** G** is the variance-covariance matrix of resistance between any pair (i,j) of herbicide treatments, *σ*(*ij*)/*σ*(*i*)*σ*(*j*), based on the observed correlations, and **β** is the vector of selection differentials imposed by a herbicide treatment (*μ*_selected_ - *μ*). Truncating selection was applied by selecting individuals with resistance value of one unit of standard deviation greater than the mean. The mean resistance level of selected individuals was defined as

(4) $$\begin{array}{c} \mu_{selected}=\frac{f\left(a\right)-f\left(b\right)}{\int_{a}^{b}f\left(x\right)dx}, \end{array}$$

where* f* is the probability density function of the standard normal distribution,* a* is the phenotypic value at +1*σ*_p_ from the mean (corresponding to the truncating selection threshold), and* b* is 100% resistance. Three herbicide application scenarios were simulated: (1) continuous single herbicide, (2) herbicide rotation (sulfometuron, terbuthylazine, glyphosate, trifluralin) assuming no correlations between herbicide responses, and (3) herbicide rotation using the observed herbicide response correlations.

Resistance development was expressed as percent change in resistance from the initial resistance level,

(5) $$\begin{array}{c} \Delta \phi =\frac{\phi_{t}-\phi_{0}}{\phi_{0}}\times 100, \end{array}$$

where Φ_t_ is the level of resistance at time* t*. The resulting resistance development curves were modelled using the logarithmic function,

(6) $$\begin{array}{c} \phi_{t}=a\log_{e}\left(t\right), \end{array}$$

and the scaling parameter* a *was transformed from logarithmic scale back into linear scale so that the unit becomes change in resistance per unit time,

(7) $$\begin{array}{c} \Delta_{\phi /t}=\frac{a}{exp\left(1\right)}. \end{array}$$

This transformed metric was used to compare the rates of resistance development under the three different herbicide application scenarios.

## 3. Results

### 3.1. Herbicide Resistance Based on Binary Dead/Alive Data

Based on binary survival data, the survival rates to the different herbicides differed significantly between populations (Table 3). All the plants were resistant to trifluralin. All the plants were susceptible to glyphosate except for a single plant in the Urana population. Both populations showed variation in resistance to sulfometuron and terbuthylazine with the Inverleigh population being significantly more resistant.

### 3.2. Performance of the Colorimetric Method, instaGraminoid

The run time for image processing and image-based metrics extraction from 1,000 jpeg images (3,264 × 4,928 pixels for a total size of 4GB) on a computer using 12 cores at 2.3GHz each, and 47GB of random access memory was ~3 hours with parallelisation and ~7 hours without parallelisation.

**Figure 3:**
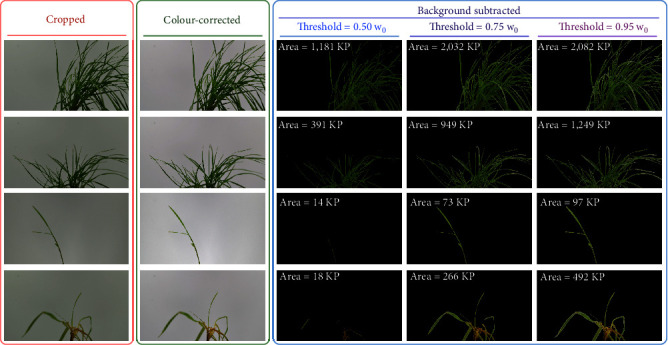
Image processing results for four sample plant photographs (w_0_ refers to the expected white background pixel value, and KP refers to ×1000 pixels).

Qualitative assessment of the image processing shows that colour correction and background subtraction using OpenCV functions were effective at detecting the above-ground plant tissues and removing the background (Figure 3). A threshold value of 75% w_0_ (75% of expected white background pixel value) enabled effective background subtraction while minimising artefacts compared with lower (50%) and higher (95%) threshold values.

**Figure 4:**
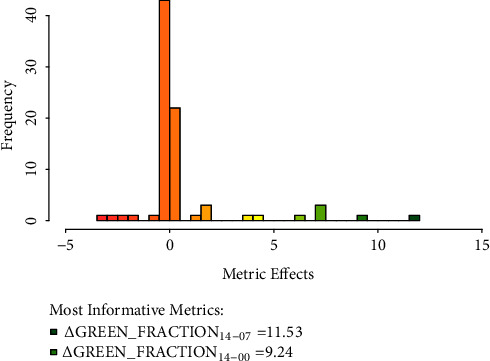
*Histogram of the metric effects used for measuring the quantitative resistance of individual plants* (*top 2 metrics are labelled* Δ*GREEN*_*FRACTION*_14-07_, change in green fraction from day 14 to day 7, and Δ*GREEN*_*FRACTION*_14-00_, change in green fraction from day 14 to day 0; uninformative features with standard deviations < 0.02 were omitted).

All the metrics extracted from the photographs were highly repeatable (low variation among technical replicates taken from different angles, ANOVA F-test p-values ≤ 7.67×10^−23^). The effects of most metrics were centred on zero and only a few metrics had large effects, which indicates an efficient sparse feature selection using elastic-net penalisation (Figure 4).

**Figure 5:**
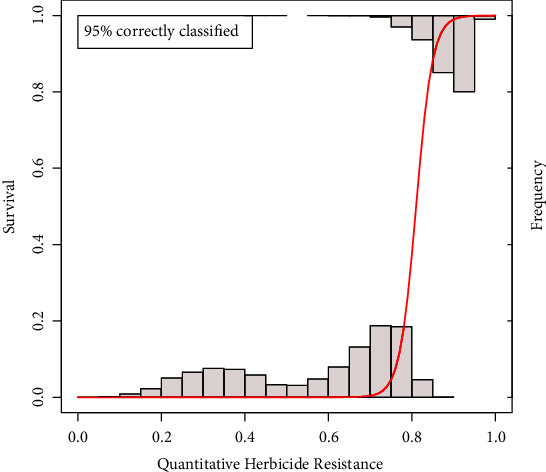
Capacity of the colorimetric model to determine resistance using logistic regression. (The histograms show the distribution of the quantitative resistance estimates in the susceptible [bottom] and resistant [top] plants. The red line is the logistic regression function relating the quantitative herbicide resistance to the observed survival data)

The* instaGraminoid* method correctly classified 95% of the plants as resistant or susceptible (Figure 5). The transferability of the phenotyping method was at least 92% across populations for a given herbicide (Table 4) and ranged from 23% to 100% across herbicide treatments (Table 5). This shows that a model trained in a single population and potentially a single herbicide can be transferred across different populations and different herbicide treatments despite different modes of action. However, models trained using glyphosate treatment data performed poorly in predicting trifluralin resistance.

### 3.3. Correlated Resistance to Herbicides

**Figure 6:**
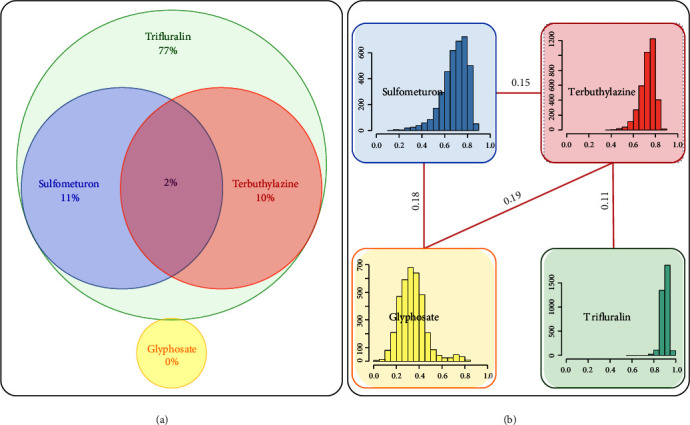
Patterns of herbicide resistance based on (a) binary survival data and (b) quantitative resistance estimates showing the significant correlations.

Measuring herbicide resistance as a quantitative trait increased the sensitivity of detecting patterns of cross-resistance. Relationships of the herbicide resistance traits were assessed as percentage of overlap using the binary survival data, as well as correlation coefficients which can be more powerfully tested (Figure 6). A number of small yet significant positive correlations were identified between herbicide pairs: trifluralin-terbuthylazine, terbuthylazine-glyphosate, glyphosate-sulfometuron, and sulfometuron-terbuthylazine, with Pearson's correlation coefficients 0.11 (p-value=0.8%), 0.19 (<0.001%), 0.18 (0.001%), and 0.15 (<0.001%), respectively.

**Figure 7:**
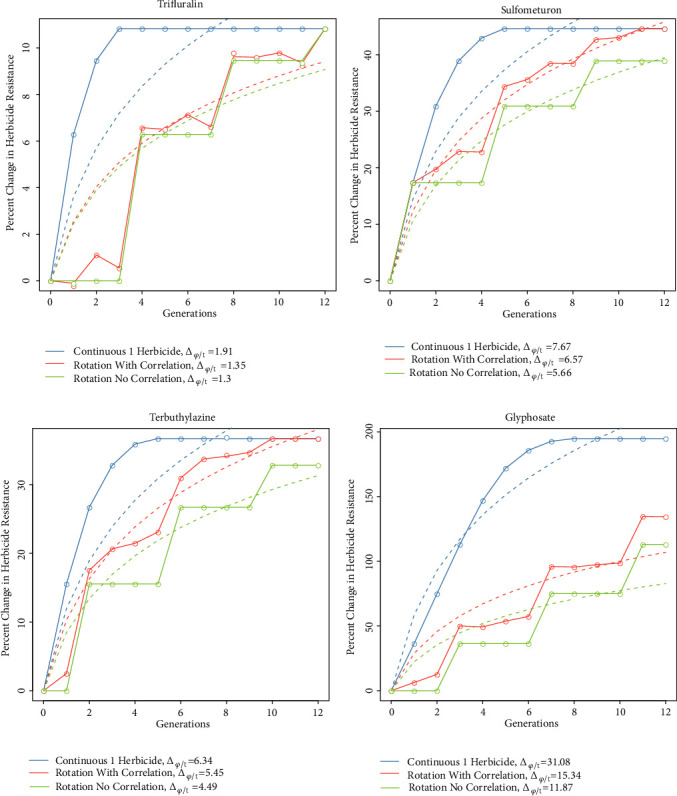
Simulated change in herbicide resistance as affected by herbicide rotation and resistance correlations (herbicide rotation: sulfometuron, terbuthylazine, glyphosate, and trifluralin).

The consequence of these subtle positive correlations in herbicide resistance on the development of resistance was simulated using a response to selection model under different herbicide rotation strategies (Figure 7). The level of resistance to a specific herbicide is predicted to increase faster under continuous application of a single herbicide compared to herbicide rotation. Resistance could fixate as early as 3 generations of continuous herbicide application for populations with high initial resistance levels as in the trifluralin treatment or as late as 7 generations for populations with low initial resistance levels as in the glyphosate treatment. The rotation strategy is predicted to be less effective in the presence of correlated resistance, with the percent change in resistance per generation (Δ_*φ*/t_) ranging from 1.30% to 11.87% in absence of correlation and from 1.35% to 15.34% in the presence of these subtle positive correlations. For a population with high initial level of resistance, there is only 0.05% difference in the change in resistance per generation between correlated and uncorrelated resistance traits (i.e., trifluralin treatment); for a population with a low starting resistance level, there is 3.47% difference in the change in resistance per generation between correlated and uncorrelated resistance traits (i.e., glyphosate treatment).

## 4. Discussion

### 4.1. Colorimetry as a Robust Approach to Measuring Plant Health

The instaGraminoid method accurately reflects plant survival after herbicide application and expresses herbicide resistance as a quantitative trait. It captures the essential image-based metrics explaining plant resistance and susceptibility to herbicides. Under stress and senescence, plants remobilise nitrogen from the photosynthetic tissues, which results in chlorophyll breakdown [31, 45, 46]. More susceptible plants undergo greater stress and experience more rapid chlorosis and necrosis. This physiological change was captured in photographs and quantified as colorimetric features or metrics. The changes in green fraction from day 7 to 14 (*ΔGREEN_FRACTION*_14-07_) and day 0 to 14 (*ΔGREEN*_*FRACTION*_14-00_) are the most informative metrics after excluding the metrics with very low variances. In more complex context, deep learning could be used to expand the set of features from the photographs [47, 48] used in the machine learning (glmnet) algorithm to predict resistance. However, this might generate biologically uninterpretable metrics and may result in parameter overfitting, thus decreasing transferability [47]. In addition, we aimed to select as model features the metrics that capture the progression of chlorosis and necrosis over time.

The rate at which chlorosis, necrosis, and eventual plant death progressed differed not only between resistant and susceptible plants but also within each of these groups. This provides a strong justification for our colorimetric approach capable of measuring quantitatively these subtle yet significant differences in resistance. This was accomplished in a resource-effective, accurate, and straightforward framework. However, when dealing with hundreds to thousands of plants, the task of photographing each individual plant is time consuming. Thus the next challenge is to extend the capability of* instaGraminoid* to identify and analyse photographs with multiple plants. In addition to the currently built-in functionalities and metrics, users can add their own metrics and use their own data to calibrate the model to specific weed species, populations, and herbicides. The script “*openCV_plant_colorimetric.py*” can be modified to add new metrics and the script “*cross_validation.r*” can be used to build, train, and test models using new input data. While the present study aimed to identify the most informative and biologically relevant metrics, user-specified metrics can be extracted to explain the traits of interest instead of applying the machine learning procedure for feature selection to explain a target trait. This default model is nonetheless highly efficient for the current application of the method and successful at defining a quantitative resistance estimate by optimising the fit to the experimental data.

We showed through cross-validation that a model trained for one herbicide is highly transferable to other herbicide treatments with different modes of action. This demonstrates that the rates of chlorosis and necrosis are generally good indicators of plant health regardless of the type of herbicide used and possibly regardless of the type of stress, biotic or abiotic. However we observed poor transferability when the model was trained on a dataset with low phenotypic variation, i.e., glyphosate treatment where most plants were susceptible. This suggests that high levels of phenotype variability in the training dataset are critical for accurate prediction. Optimal transferability of the model can be achieved by training on a dataset representing the maximum range of phenotypic variation [49]—this applies to both the metrics and the response variable, whether it is herbicide resistance or any other plant health-related trait. The main recommendation for accurate prediction and early plant health assessment is to ensure that maximally variable populations are sampled for model training.

### 4.2. Relevance of the Method to Identify the Molecular Basis of Herbicide Resistance

Expressing herbicide resistance of individual plants as a quantitative trait using predictive models will enable fine-level and near real-time monitoring of plant health. Using our colorimetric method* instaGraminoid *and pretrained* glmnet *predictive models, herbicide resistance of individual plants can be detected early using photographs.

Of the four herbicides used in this study, two resulted in low resistance variability in the two populations tested. These are glyphosate and trifluralin. Most of the plants did not survive 21 days after glyphosate application, most likely due to the low frequency of resistance-conferring alleles in the two populations. In comparison, all the plants survived trifluralin application. This apparent resistance of all the plants to trifluralin may be attributed to insufficient dose and application via spraying instead of the recommended soil-incorporation method [50]. This probably resulted in high volatilisation of the herbicide and low absorption efficiency by the root and shoot apical meristems [51]. Trifluralin is used as a preemergence soil-incorporated herbicide, and the recommended dosage used was optimised for germinating seeds not plants at mid-vegetative or tillering stage used in this study. However, the use of mid-vegetative plants was imperative to compare clones and test cross- and multiple resistance, and similar postemergence application of trifluralin has been shown to cause growth retardation in cotton plants when the terminal buds were exposed [52]. An alternative would be to use families (half-sibs, full-sibs, early selfed, or populations) but the one-to-one correspondence will be confounded by the heterogeneity of genotypes sampled across replications and treatments, thus requiring larger sample sizes to minimise variance within group.

The quantitative herbicide resistance estimates will enable the application of powerful statistical analysis of the genetic basis of resistance, the accurate modelling of herbicide resistance evolution in agroecosystems, and ultimately the improvement of herbicide resistance management strategies. Binary survival data has been sufficient for studying the genetic basis of target-site mutations and has been done extensively for many species and herbicides [17, 53–57]. However, the search space for the causal genetic variants has typically been limited to the target-site genes and well known detoxifying genes such as cytochrome P450 monooxygenases and glutathione S-transferases. Direct genetic studies without prior assumptions of the genetic elements conferring resistance such as genome-wide association studies are yet to be implemented in weeds. Our approach paves the way for harnessing high-throughput phenomics to next generation genomics in weeds.

### 4.3. Applications to Field Management of Resistant Weeds

The colorimetric method for herbicide resistance estimation presented here is more resource-effective compared to existing alternatives. A simple RGB camera using visible light sensors with resolutions of at least 5 megapixels can be used. In its current implementation, the method has limited portability; however, it can be used in the field with pictures of the plants taken* in situ* by adapting the background subtraction function to isolate the plant tissues from the complex background. Any plant stage should be amenable to our protocol as long as the plants are not yet senescent as a result of natural aging and the level of stress is enough to induce a response in susceptible plants (e.g., the herbicide can be absorbed by the plant and the dosage is enough to induce plant stress responses). However hyperspectral imaging may outperform our method because of the massive amounts of predictors that can be acquired, but the initial investment for acquiring the equipment is high. These can be used in a wide range of applications requiring noninvasive and nondestructive measurements. Abiotic and biotic stresses in plants have been measured using NIR spectroscopy [27].

Nonetheless, the* instaGraminoid* method enabled a direct estimation of cross-resistance. Pairwise resistance correlations between trifluralin-terbuthylazine, terbuthylazine-glyphosate, glyphosate-sulfometuron, and sulfometuron-terbuthylazine treatments were observed. These significant positive correlations in herbicide resistance are indicative of the absence of trade-offs in resistance across herbicides with different modes of action. The correlated resistance development shown by the herbicide rotation simulations depends on the assumption that the major genetic basis of resistance is NTSR. This needs to be validated through GWAS and functional genetics studies to confirm the genes/regulatory elements controlling resistance.

Heterogeneous herbicide applications across time and space (i.e., combination and rotation of different herbicides) are recommended to delay or manage the evolution of herbicide resistance [58–60]. However, the effectiveness of this strategy assumes independent, herbicide-specific TSR genes. In the presence and predominance of cross-herbicide NTSR genes, this strategy may be ineffective or even counterproductive. Under this strategy, the development of target-site resistance would be delayed but the development of non-target-site resistance that can induce resistance to multiple herbicides may be hastened, which will be more problematic. Weed populations may develop resistance to herbicides they have not been previously exposed to. Integrated Weed Management (IWM) has been designed as a systems approach to minimising not only yield losses but also the negative impacts on the environment and health by managing weed populations [61]. IWM promotes the heterogeneity of weed control strategies, not just chemical control, but also mechanical, physical, and biological weed control methods. The overreliance on herbicides has promoted the evolution of herbicide resistant weed populations; however, herbicides remain to be the corner stone of modern weed control. In this context, timely monitoring of resistance levels through phenomics assays or genomic prediction (i.e., prediction of resistance levels based on genomic information) will boost the effectiveness, efficiency, and sustainability of integrated weed control strategies.

## 5. Conclusion

The* instaGraminoid* method (https://gitlab.com/jeffersonfparil/instaGraminoid.git and https://gitlab.com/jeffersonfparil/instaGraminoid_executables.git) will provide end-users with a new, accurate, repeatable, and transferable herbicide resistance assay that can be generalised for other types of plant health assessment. This method is able to quantify herbicide resistance of individual plants efficiently and accurately using visible light imaging and image analysis. Leaf greenness metrics were the most informative colorimetric features across the four herbicide treatments. The calibration of the model using* elastic-net *penalisation in a generalised linear model framework enables high transferability across populations and herbicide treatments. Using the quantitative herbicide resistance estimates, significant positive correlations were observed across herbicide treatments which may exacerbate the evolution of cross-resistance.

## Figures and Tables

**Table 1:** List of herbicides used to characterise herbicide resistance in two Lolium rigidum populations.

HRAC Group	Commercial Product Name	Active Ingredient (a.i.)	Recommended Field Rate (g a.i. ha^−1^)
B	Apparent Sulfometuron 750WG	Sulfometuron	400
C	Nufarm Terbazine 875WG	Terbuthylazine	1,050
G	Yates Weedkiller 490SC	Glyphosate	720
K1	Nufarm TriflurX 480EC	Trifluralin	816

**Table 2:** Plant health metrics derived from photographs of above-ground tissues of Lolium rigidum plants.

Metric	Description
*a*	leaf area visible from one angle (1 angle: 1 technical replicate)
*μ*_*B*_	mean pixel value in the blue channel (range 0-255)
*μ*_*G*_	mean pixel value in the green channel (range 0-255)
*μ*_*R*_	mean pixel value in the red channel (range 0-255)
*m*_*B*_	median pixel value in the blue channel
*m*_*G*_	median pixel value in the green channel
*m*_*R*_	median pixel value in the red channel
*GREEN_INDEX*	$\left[\left(1+\frac{\mu_{G}-\mu_{R}}{\mu_{G}+\mu_{R}}\right)+\left(1-\frac{\mu_{B}+\mu_{G}+\mu_{R}}{3\times 255}\right)\right]$
*GREEN_FRACTION*	$\frac{\mu_{G}}{\mu_{B}+\mu_{G}+\mu_{R}}$
*P*(*H*_*B*_)	density-derived frequency of blue pixel values between 0 and 25
*P*(*H*_*G*_)	density-derived frequency of green pixel values between 100 and 125
*P*(*H*_*R*_)	density-derived frequency of red pixel values between 66 and 80
*T*_*G*1_	proportion of green pixel values greater than 100
*T*_*G*2_	number of green pixel values greater than 100 divided by the number of green pixel values less than 100
*T*_*R*1_	proportion of red pixel values greater than 150
*T*_*R*2_	number of red pixel values greater than 150 divided by the number of green pixel values less than 150
Δ_i_	change in the above metric values between two measurement times, i.e., Day 14-Day 7, Day 14-Day 0, and Day 7-Day 0

**Table 3:** Proportion of resistant plants to the four herbicides. Chi-square test (df=1) was applied to test for independence between the two populations.

Herbicide	Inverleigh	Urana	*χ* ^2^ test p-value
Trifluralin	1.0000	1.0000	NA
Sulfometuron	0.1826	0.0567	9.21 × 10^−9^
Terbuthylazine	0.1585	0.0452	3.13 × 10^−8^
Glyphosate	0.0000	0.0023	9.65 × 10^−1^

**Table 4:** Proportion of plants correctly classified using the instaGraminoid colorimetric models trained on the populations in the row and validated in populations in the column (all these correlations are different based on Tukey's honest significance difference grouping at *α*=0.05).

Training Population	Validation Population	
	Inverleigh	Urana
Inverleigh	0.9347	0.9700
Urana	0.9201	0.9732

**Table 5:** Fraction of entries correctly classified using the instaGraminoid colorimetric models trained on herbicide treatments in the row and validated in herbicide treatments in the column (the letters correspond to Tukey's honest significance difference grouping at *α*=0.05).

Training Herbicide Treatment	Validation Herbicide Treatment			
	Trifluralin	Sulfometuron	Terbuthylazine	Glyphosate
Trifluralin*∗*	-	-	-	-
Sulfometuron	0.8732^e^	0.9378^b^	0.8755^e^	0.9989^a^
Terbuthylazine	0.8892^d^	0.9016^c^	0.9082^c^	0.9989^a^
Glyphosate	0.2314^g^	0.8665^f^	0.8858^d^	1.0000^a^

*∗* Model training using response variable data without any variation is not possible; i.e., all the plants were unaffected by the trifluralin treatment.
